# Ecosystem services for intensification of agriculture, with emphasis on increased nitrogen ecological use efficiency

**DOI:** 10.1002/ecs2.3028

**Published:** 2020-02-07

**Authors:** Virley G. L. Sena, Emanoel G. de Moura, Vinícius R. A. Macedo, Alana C. F. Aguiar, Adam H. Price, Sacha J. Mooney, Juliano C. Calonego

**Affiliations:** ^1^ Department of Crop Science College of Agricultural Sciences São Paulo State University Botucatu São Paulo 18.610‐307 Brazil; ^2^ Postgraduate Program in Agroecology Maranhão State University São Luis Maranhão 65000‐000 Brazil; ^3^ Federal Institute of Education, Science and Technology of Piauí Uruçuí Piauí 64860‐000 Brazil; ^4^ Department of Biology Federal University of Maranhão São Luís Maranhão 65080‐805 Brazil; ^5^ Institute of Biological and Environmental Sciences University of Aberdeen Aberdeen AB24 3UU UK; ^6^ School of Biosciences University of Nottingham Sutton Bonington Campus Loughborough LE125RD UK

**Keywords:** nutrients recycling, soil organic matter, soil rootability, sustainability

## Abstract

In weathered tropical soil, low nutrient use efficiency can lead to agricultural systems becoming unsustainable. Therefore, tropical agriculture is highly dependent on ecosystem services, such as nutrient recycling and carbon sequestration, to enhance soil fertility, increase nutrient uptake, and facilitate sustainable production of agricultural goods. This research aimed to find the balance between sustainability and profitability of tropical agriculture by evaluating the changes in soil caused by the ecosystem services provided by the biomass of leguminous trees (*Gliricidia*) and assessing how these changes (associated with potassium) can affect nitrogen‐use efficiency and maize yield. An experiment was conducted testing the impact of *Glircidia* biomass addition vs. bare soil, with or without addition of both nitrogen and/or potassium. Changes in soil organic matter, (SOM) base cations sum, soil resistance, N uptake, N‐use efficiency, and maize yield were evaluated. Gliricidia biomass, when used with N and K, contributed to increasing SOM by 5.0 g/kg and the sum of base cations by 1458. 65 kg/ha in the 0–30 cm layer. Moreover, grain yield was increased by approximately 70% in the treatments with *Gliricidia* when compared to treatments without biomass where yield was very low. In bare soil, the additional yield of 1.5 tons/ha would not be enough to convince farmers to change slash and burn to conventional bare soil systems. Our results showed that leguminous trees, such as Gliricidia, might contribute to ensuring sustainable agricultural intensification in humid tropical soils with low natural fertility by providing ecosystem services such as biomass production, carbon sequestration, base cation recycling, and increased N acquisition. These findings might be an important strategy to replace the common slash‐and‐burn‐system and preserve the rainforest against the traditional shifting cultivation system. In contrast, the conventional system with bare soil showed that the addition of nitrogen was unfeasible, mainly in conditions of high rainfall precipitation. In these circumstances, the use of potassium may increase nitrogen‐use efficiency only when biomass is not used.

## Introduction

Agricultural products are essential to human well‐being, but the sustainable production of agricultural goods is highly dependent on the services provided by neighboring natural ecosystems (Malhi 2012). Preliminary assessments indicate that the value of these ecosystem services to agriculture is enormous and often underappreciated (Power 2010). In weathered tropical soil, the services provided by natural ecosystems, such as nutrient cycling and increased carbon sequestration in the soil root zone, is essential to the maintenance of soil structure and the enhancement of soil fertility. These processes drive the efficiency by which plants acquire the major nutrient nitrogen (N), which has been identified as one of the most important targets for improving root acquisition efficiency (Chapman et al. 2012). This is particularly true in humid tropical conditions, where nitrogen‐use efficiency is generally very low. Primarily, high temperatures increase volatilization rates from urea (Viero et al. 2014), while high rainfall rates increase nitrate concentration and leaching (Jabloun et al. 2015). In addition, in tropical soil containing very low levels of free iron and organic carbon, the rootable soil volume may be decreased during drying soil impairing nutrient uptake (Moura et al. 2018). Therefore, the inefficiency of nitrogen use, beyond having a negative economic impact, contributes to agriculture disservices by greenhouse gas emissions and pollution of groundwater (Rutting et al. 2018). Thus, nitrogen uptake that is impaired in conditions of both high rainfall (by nitrogen removal) and low rainfall (by reduced root growth in hardsetting soil. Thus, irregular precipitation rates occurring where soils undergo hardsetting during drying cycles can result in nitrogen uptake that is impaired in conditions of both high rainfall (by nitrogen removal) and low rainfall (by reduced root growth in hardsetting soil; Moura et al. 2018).

In the circumstances above, as reported by Moura et al. (2013), a resulting nitrogen agronomic efficiency as low as 14 kg/kg (maize grain/N applied) has been the main reason that many family farmers have resisted changing from the traditional slash‐and‐burn systems to conventional tillage systems, despite recommendations to switch. In addition, because of a rule specifying that just 20% of a farmer's area can be used for agriculture in the Amazonian region, emerging local research and policy agendas now demand other agricultural systems, based on sustainable intensification, for which increased nutrient use efficiency is crucial (Zhang et al. 2017).

To increase nitrogen‐use efficiency, primary measures should include improvement in soil rootability to increase rot growth (Rutting et al. 2018). Strategies to enhance soil rootability and increase nutrient uptake include the following: (1) appropriate use of mulching; (2) application of biomass to increase soil organic matter; (3) addition of base cations, such as calcium and magnesium; and (4) establishment of adequate soil ionic balance.

Mulches may delay hardsetting and favor root growth by reducing evaporation from the soil surface (Mulumba and Lal 2008). In addition, the continuous application of biomass, as occurs with mulching, may increase the labile fractions of organic matter, thereby functioning in creating aggregates that, although unstable, improve the porosity and thus soil environment for root growth (Passioura 1991).

To extend rootability to lower soil depths, previous research has suggested using calcium and magnesium as “flocculating agents” to improve soil structure by reducing the dispersion of the clay (Anikwe et al. 2016). In turn, the ionic balance has been identified as having an effect on nitrogen uptake. Fenn and Taylor (1988) reported the possibility of calcium (Ca)‐stimulated ammonium (NH_4_) uptake by plants. Field studies revealed significant increases in plant growth due to an increase in the Ca:NH_4_ ratio. In addition, previous research has also reported a positive influence of potassium on N uptake (Khalifa et al. 2012). An ample supply of potassium to plant roots counteracts a possible toxic effect of ammonium nutrition, enhancing ammonium utilization.

Unfortunately, in tropical regions, accumulation of organic matter, which could mitigate the negative effects of cohesion on soil rootability, is impaired by conditions that favor rapid decay of incorporated biomass (Hijbeek et al. 2018). On the other hand, it has been shown that organic matter may be retained in the solid phase as complexes bound together by protons and divalent ions, mainly calcium and magnesium (Whittinghill and Hobbie 2012, Ellerbrock and Gerke 2018). Therefore, the ionic composition of the soil solution influences organic matter state, particularly coagulation/dissolution (Oste et al. 2002). The interactions between calcium, magnesium, and organic compounds derived of biomass decomposition can result in greater permanence of both calcium and magnesium in the soil, in turn improving the soil rootability and conditions for root growth (Moura‐Silva et al. 2017).

In the humid tropics, despite great constraints to crop productivity, there are some environmental advantages, such as high soil moisture paired with consistent year‐round warm weather, which provides appropriate conditions for growth of adapted vegetal species (Berenguer et al. 2018). Indeed, it has been reported that the use of biomass produced by leguminous trees in alley cropping system has shown great potential as a scalable agricultural alternative that can enhance production while simultaneously improving sustainability (Moura et al. 2018, Aguiar et al. 2019). In these circumstances, high growth capacity of leguminous trees, if used wisely, can provide ecosystem services such as biomass production, recycling of nutrients, nitrogen fixation, and carbon sequestration (Medinski and Freese 2012). These services are essential to enhance soil fertility, increase crop productivity, and ensure sustainability of tropical agroecosystems (Muller et al. 2017). The tree arrangement in alley cropping facilitates management practices that reduce competition and increase complementarity between trees and crops. In addition, if used this way alley cropping offers the advantage of bringing together, in the same space and at the same time, crop cultivation and soil fertility regeneration (Moura et al. 2013).

Therefore, given the local circumstances, where both unfavorable and propitious conditions are present and acting simultaneously, we hypothesized that these favorable factors (biomass production, recycling of nutrients, nitrogen fixation, and carbon sequestration), if used wisely, could allow for the establishment and management of an integrated crop‐tree‐system, thereby overcoming the adversities. The confirmation of this hypothesis could ensure reconciliation between sustainability and profitability of tropical agriculture by establishing the benefit of ecosystem services provided by leguminous trees. Therefore, the aim of this study was to evaluate the changes in soil fertility and nitrogen uptake caused by the ecosystem services provided by leguminous biomass and how these changes can contribute to achieving sustainable agricultural intensification increasing nitrogen‐use efficiency and maize yield, when associated with potassium.

## Materials and Methods

### Characterization of the experimental area and experimental setup

**Figure 1:**
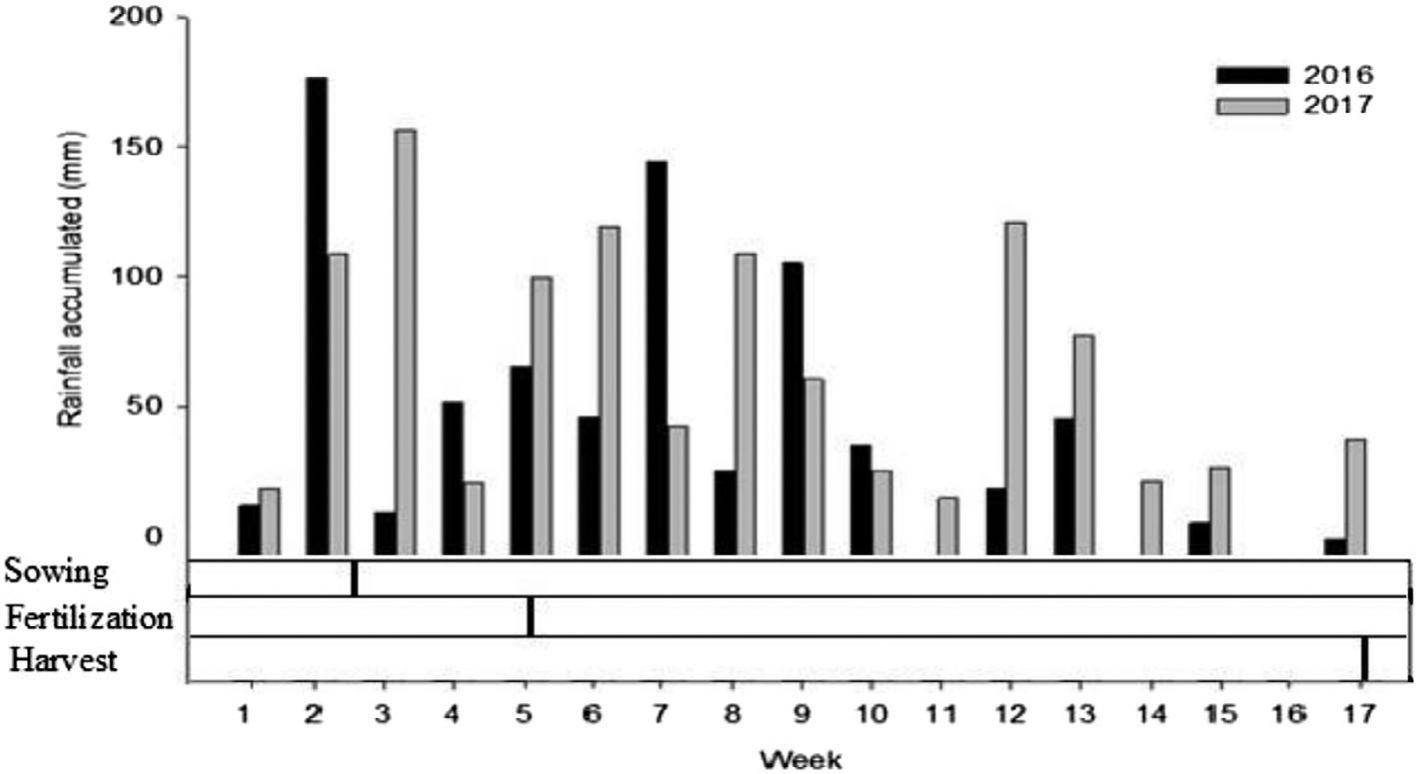
Timeline showing rainfall, sowing, fertilizer application and harvest period.

Experimental work was conducted in the county of Brejo‐MA, Brazil, 3°39′ S latitude and 42°57′ W longitude. The climate of the region, according to Köppen, is Aw type, hot and humid equatorial, and the local average annual rainfall was 1100 mm. The precipitation during the experiment period was 816 mm in 2016 and 1112.4 mm in 2017 (Fig. 1). The soil of the study area was classified as Arenic Hapludults (Soil Survey Staff 2010) and displayed hardsetting characteristics (Moura et al. 2009), with an A horizon of the following properties: pH of 4.2 (in CaCl_2_); 25 g/kg of organic carbon; 34.5 mg/dm^3^ of P; 51.0 mmol_c_/dm^3^ of (Al + H); 19.6 mmol_c_/dm^3^ of Ca; 3.8 mmol_c_/dm^3^ of Mg; 0.8 mmol_c_/dm^3^ of K; 75.3 mmol_c_/dm^3^ of capacity of exchange cation (CEC); 32.2% of the base saturation percentage; 280 g/kg of coarse sand, 520 g/kg of fine sand, 100 g/kg of silt; and 100 g/kg of clay.

### History and conduct of the experimental area

In January 2011, the area, which had been in fallow since 2008, was treated after rice cultivation with surface limestone and gypsum applied at a rate of 2 t/ha of hydrated calcium and 6 t/ha, respectively. The application rates of lime and gypsum were calculated to raise base saturation to 55% and sum of bases to 45 mmol_c_/dm^3^ in the 0–30 cm layer. The leguminous tree *Gliricidia sepium* was used in an modified alley cropping system where trees are pruned annually and biomass applied on soil surface. Gliricidia was sown in January 2011 with spacing of 0.5 m between trees and 4.0 m between rows. The area was divided into four equal blocks of 320 m² each, within which were eight plots of 10 × 4 m. In the years 2013, 2014, and 2015, maize (*Zea mays* L.) was cultivated during the rainy season among the rows of legumes. In each year, the soil was fertilized with 120 kg/ha of P_2_O_5_ in the form of triple superphosphate, 60 kg/ha of K_2_O as KCl, 60 kg/ha of N in the form of urea, and 5 kg/ha of Zn in the form of zinc sulfate. The first pruning of the gliricídia was carried out in 2013 and continued in the following years. The annual pruning was 0.5 m above ground level, and the green biomass of the legume was distributed evenly between the maize lines shortly after planting. The amount of leguminous biomass applied was 3.5, 6.2, and 7.8 tons/ha of dry matter in 2013, 2014, and 2015, respectively.

### Experimental design this experiment

In January 2016 and 2017, at the start of the rainy season the non‐selective action herbicide glyphosate was applied with a costal sprayer to control the weeds in the experimental area. Maize (*Zea mays* L. cv Ag 1051) was sown between the rows of Gliricidia, establishing four rows spaced 80 cm apart and with 25 cm between plants. A randomized block design was used in 4 × 10 m plots with four replications and the following 8 treatments: (1) Gliricidia with 60 kg/ha of K, 60 kg/ha of N (GKN); (2) Gliricidia with 60 kg/ha of N (GN); (3) Gliricidia with 60 kg/ha of K (GK); (4) Gliricidia (G); (5) bare soil, 60 kg/ha of K, 60 kg/ha of N (BSKN); (6) bare soil 60 kg/ha of N (BSN); (7) bare soil 60 kg/ha of K (BSK); and (8) bare soil (BS). All treatments received 120 kg/ha of P_2_O_5_ as triple superphosphate and 5 kg/ha of Zn in the form of zinc sulfate. The N application as urea was divided into 15 kg/ha applied at planting and 45 kg/ha applied 35 d after maize planting. The amount of dry matter of leguminous biomass applied was 5 tons/ha (4% N), consisting of 200 kg/ha of organic N in the form of *Gliricidia sepium* in the treatments previously determined to receive biomass.

### Soil chemical properties

To carry out the analyses of soil chemistry, physical fractionation of soil organic matter and soil particle size distribution, in May 2016 and 2017, the soil samples were collected with a probe‐type auger. A total of nine single samples per plot were collected to obtain a composite sample at depths of 0–10 and 10–30 cm. Samples from each plot were air‐dried and passed through a 2‐mm sieve before analysis.

The soil organic matter was physically fractionated, according to the methods of Cambardella and Elliot (1992). Air‐dried soil samples of 20 g were sieved through 2‐mm mesh and weighed in 250‐mL polyethylene cups, to which 80 mL of 5 g/L sodium hexametaphosphate was added. The mixture was shaken for 15 h in a horizontal shaker, with 130 oscillations per min. After this process, the entire contents of the vial were placed into a 0.053‐mm mesh sieve and washed with a weak jet of distilled water until the clay was completely removed. The material retained on the sieve was defined as total particulate organic matter (>53 μm) and was dried at 50°C. After drying, the sample was ground in a porcelain mortar, following which an aliquot was collected, weighed, and analyzed for its C content, representing the soil particulate organic carbon (POC) in particulate organic matter, according to the Walkley‐Black method. An aliquot of the 2‐mm sieved subsample was ground in a porcelain mortar and weighed and analyzed for the analysis of soil total organic carbon (TOC). Soil mineral‐associated carbon (MOC) was calculated as the difference between TOC and POC. The total organic carbon stock (TOCS) of each of the 0–30 cm layers was calculated by the following expression (Veldkamp 1994): C stock = (TOC × ρs × *E*)/100, where C stock = organic C stock at a given depth (Mg/ha); OC = organic C content at the sampled depth (g/kg); ρs = soil bulk density (kg/dm^3^); and *E* = thickness of the layer (30 cm). Accumulated organic matter was calculated as the difference between TOC of the treatment and TOC of the control (BS).

The soil chemical analyses (0–30 cm) were as follows: K^+^, Ca^2 +^, and Mg^2 +^. Each sample was analyzed for exchangeable K, Ca, and Mg using an “exchangeable ion resin” (Raij et al. 1986). Ca, Mg, and K measurements were obtained using a Varian 720‐ES ICP Optical Emission Matter Analysis Spectrometer. The accumulations of Ca, Mg and K, in kg/ha, in the 0–30 cm layer of the soil profile, were calculated according to the equation of Ellert and Bettany (1995): SEA = SEC × ρs × *E* × 10, where SEA = soil element accumulation (kg/ha); SEC = soil element content (mg/kg); ρs = soil bulk density (Mg/m^3^); and *E* = thickness of the layer (m). The accumulation of cationic bases (ACB, in kg/ha) was calculated by the difference means between treatments—the sum of Ca, Mg, and K accumulations in the control.

### Soil physical properties

Undisturbed soil samples, collected in May 2017, in volumetric rings with a 100‐cm^3^ volume to determine soil bulk density (ρs), data necessary to calculated soil carbon stocks. Soil bulk density (ρs) was calculated as m/v, where m is the dry collection soil mass at 105°C and v is the ring volume (Thomasson 1978). Three replicates were collected per section at depths 12–20 cm. The soil moisture and soil penetration resistance were measured at depths of 0–5, 5–10, 10–15, and 15–20 cm, with three replicates per plot, after 5 d without rain. The soil penetration resistance was measured using a digital penetrometer (Falker, Porto Alegre, Brazil) with 1 cm gradations. To calculate soil moisture content by gravimetric method, the following was used: soil moisture (g/g) = weight of fresh soil − weight of oven‐dried soil/weight of oven‐dried soil.

### Plant analysis

Plant dry matter and total nitrogen content were measured in two physiological periods of maize: at tasseling and at the physiological maturity stage. At each sampling, five plants from each plot were randomly selected. All plant materials were dried at 60°C for 3–4 d to obtain a constant weight and were ground for analysis. The amount of nitrogen in each sample was determined from the mass of dried plant matter by digestion in sulfuric acid (H_2_SO_4_–H_2_O_2_), according to the standard distillation method described by Cottenie (1980). The various parameters related to nitrogen translocation in the maize plant were calculated according to the following formulas: 1, 2, 3, 4, and 5 according to Fageria and Baligar (2005):

(1) $$\text{Accumulated nitrogen at tasseling}\left(\text{ANAT}\right)=\frac{\text{dry matter}(\text{kg}/\text{ha})\times \text{N at tasseling}(g/\text{kg})}{1000}$$

(2) Accumulated nitrogen post‐tasseling(ANPT)=total nitrogen(kg/ha)−N at tasseling(kg/ha)

(3) Total accumulated nitrogen(TAN)=ANAT−ANPT

(4) $$\text{Nitrogen recovery efficiency}\left(\text{NRE}\right)=\frac{\text{N uptake in treatment}(\text{kg}/\text{ha})-\text{N uptake in control}(\text{kg}/\text{ha})}{\text{Total applied mineral N}(\text{kg}/\text{ha})}\times 100$$

(5) $$\text{Nitrogen agronomic efficiency}\left(\text{NAE}\right)=\frac{\text{kg crop yield in the treatment}-\text{kg crop yield in the control}}{\text{N applied}(\text{kg}/\text{ha})}$$

### Statistical analyses

The data were analyzed using ESTATISTIX program (version 9). All data were submitted to analysis of variance (ANOVA), and the means were compared statistically by Fisher's LSD test at a level of 5% probability of error**.** The software SIGMAPLOT (version 11.0) was used to plot the graphs.

## Results

### Soil penetration resistance, soil moisture, and N uptake

**Figure 2:**
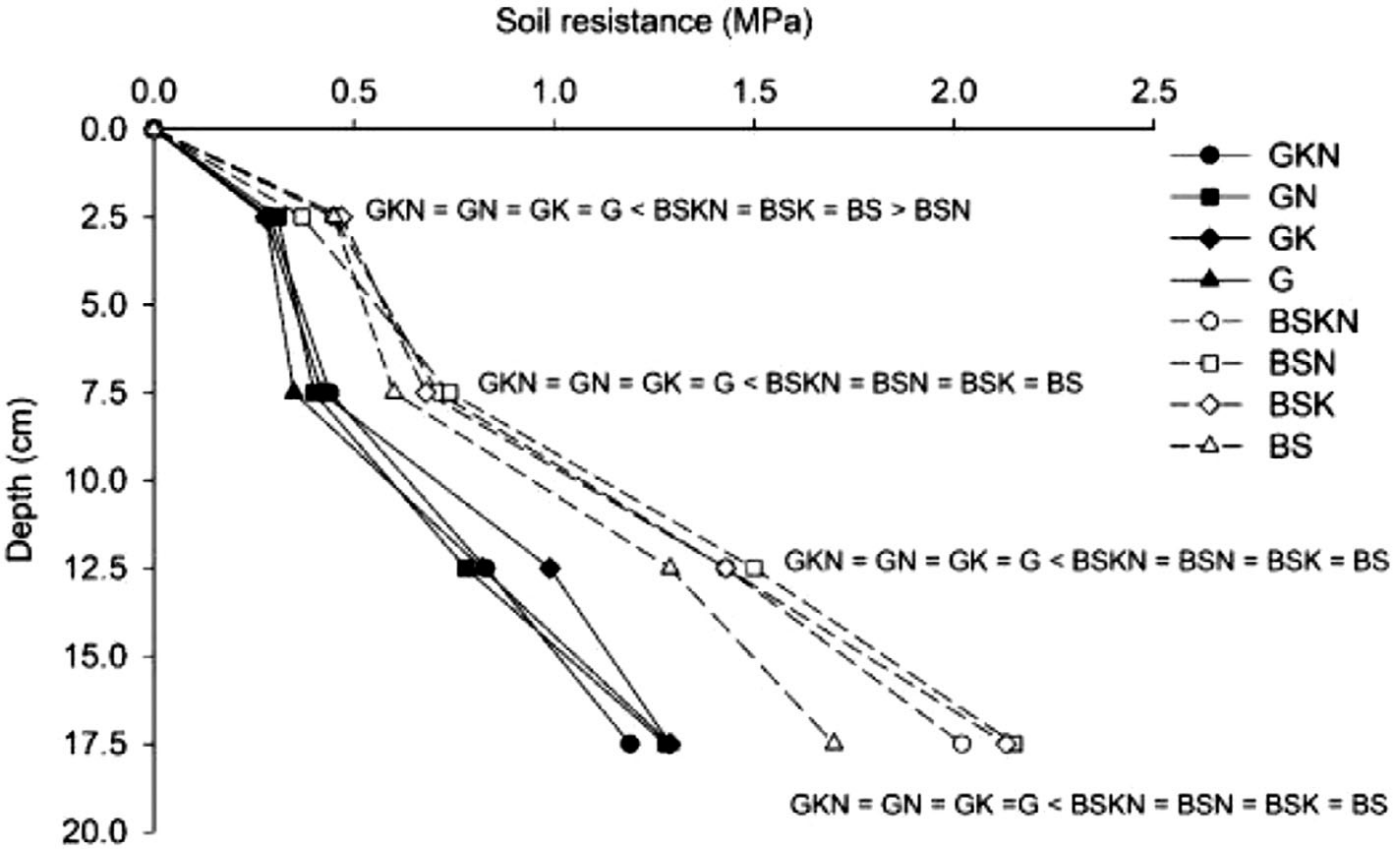
Soil penetration resistance (MPa) after 7 d without rain, at 0–20 cm layer.

Biomass decreased the soil penetration resistance at all depths evaluated. Larger differences were found in the deeper layer (15–20 cm) than in the 0–5 cm layer (Fig. 2). There was not any effect of N and K application on soil penetration resistance. Soil moisture was greater in covered treatments up to the 0–10 cm layer. In the 10–20 cm layer, the differences in soil moisture between treatments covered and uncovered were statistically insignificant (Table 1).

**Table 1:** Average soil moisture content for the experimental treatments, in the depth of 0–5, 6–10, 11–15, and 16–20 cm

Treatments	Soil moisture (g/g)			
	0–5 cm	6–10 cm	11–15 cm	16–20 cm
GKN	0.17 ab	0.17 a	0.15 a	0.14 ab
GN	0.19 a	0.17 a	0.15 a	0.15 a
GK	0.19 a	0.17 a	0.15 a	0.15 a
G	0.19 a	0.17 a	0.15 a	0.15 a
BSKN	0.14 bc	0.13 b	0.13 ab	0.13 b
BSN	0.15 bc	0.13 b	0.11 b	0.14 ab
BSK	0.13 c	0.13 b	0.13 ab	0.13 b
BS	0.14 c	0.13 b	0.15 a	0.14 ab
CV	10.49	6.45	15.00	9.00

Note

Values followed by different letters in the same column indicate a significant difference at the 5% level by LSD test. GKN, Gliricidia with 60 kg/ha of K, 60 kg/ha of N; GN, Gliricidia with 60 kg/ha of N; GK, Gliricidia with 60 kg/ha of K; G, Gliricidia; BSKN, bare soil, 60 kg/ha of K, 60 kg/ha of N; BSN, bare soil 60 kg/ha of N; BSK, bare soil 60 kg/ha of K; BS, bare soil; and CV, coefficient of variation.

### Exchangeable base cation recycling

Calcium was increased by leguminous biomass and N application (GNK and GN treatments) in the 0–30 cm layer (Table 2). In the same way, magnesium was greater in treatments with biomass and N application (such as in GNK); magnesium was greater in these treatments than in all others. However, potassium content was not affected by biomass, but only by potassium application, when biomass and N were used (GKN > GN). Compared with the control (BS) treatment, the GKN treatment increased the sum of base cations in the 0–30 cm layer by 1458 kg/ha. Most of this contribution was attributed to increased calcium since the contribution of magnesium and potassium was small. When compared with BSKN, the increase in the sum of base cations provided by biomass only was 1055.77 kg/ha. In the treatments without N (GK, G), the addition of biomass did not result in a sum of base cations different from the BSKN treatment.

**Table 2:** Exchangeable base cations, sum of base (SB), and accumulated basic cations (ABC) at 0–30 cm layer

Treatments	(mmol_c_/m^3^)				(kg/ha)				
	Ca	Mg	K	SB	Ca	Mg	K	SB	ABC
GKN	29.42 a	14.92 a	1.85 a	46.17 a	2647.80 a	815.75 a	324.68 a	3788.23 a	1458.63 a
GN	27.92 a	11.83 b	1.10 b	41.85 a	2512.80 a	646.81 b	193.05 b	3352.66 b	1023.06 b
GK	21.67 b	11.00 b	1.40 ab	34.07 b	1950.30 b	601.43 b	245.70 ab	2797.43 c	467.83 c
G	22.25 b	11.18 b	1.47 ab	34.90 b	2002.50 b	611.27 b	257.99 ab	2871.76 c	542.16 c
BSKN	22.00 b	9.75 c	1.25 ab	33.00 b	1980.00 b	533.08 c	219.38 ab	2732.46 c	402.86 c
BSN	18.75 c	6.75 d	0.85 b	26.35 c	1687.50 c	369.06 d	149.18 b	2205.74 d	…
BSK	21.50 b	8.25 c	1.05 b	30.80 b	1935.00 b	451.07 c	184.28 b	2570.35 cd	240.75 c
BS	19.92 bc	7.25 cd	0.80 b	28.10 bc	1792.80bc	396.39 cd	140.40 b	2329.59 cd	…

Note

Values followed by different letters in the same column indicate a significant difference at the 5% level by LSD test. GKN, Gliricidia with 60 kg/ha of K, 60 kg/ha of N; GN, Gliricidia with 60 kg/ha of N; GK, Gliricidia with 60 kg/ha of K; G, Gliricidia; BSKN, bare soil, 60 kg/ha of K, 60 kg/ha of N; BSN, bare soil 60 kg/ha of N; BSK, bare soil 60 kg/ha of K; and BS, bare soil. Ellipses indicates 5% level by LSD test.

### Organic carbon and carbon recycling

For the treatment of bare soil plus potassium (BSK) as well as the treatment for bare soil (BS), both biomass and nitrogen application increased total organic carbon (TOC) fractions in the 0–10 cm layer (Table 3). However, TOC was greater in treatments with biomass than in treatments with nitrogen in bare soil. In the same way, biomass and nitrogen together increased organic carbon fractions in the 10–30 cm layer, in such a way that total organic carbon was greater in GKN and GN treatments and lower in BSN, BSK, and BS treatments. However, when potassium and nitrogen were used in bare soil, TOC was equal to that of treatments with biomass without nitrogen (BSKN = G, GK). The mineral organic matter fraction was most responsible for these differences. Total stock of carbon was 30% higher in treatments with biomass and nitrogen than in treatments of bare soil without N. Thus, compared with the control treatment, the treatment of GKN, when added to accumulated organic content, resulted in an additional 5.0 g/kg of total stock of carbon. Similarly, compared with the control treatment, the treatment of GN, when added to accumulated organic content, resulted in an additional 4.7 g/kg of total stock of carbon.

**Table 3:** POC, particulate organic carbon MOC, associated mineral organic carbon; TOC, total organic carbon; TOCS, total organic carbon stock; ASOM accumulated soil organic matter

Treatments	0–10 cm			10–30 cm			0–30 cm	
	POC	MOC	TOC	POC	MOC	TOC	TOCS	ASOM
	(g/kg)	(g/kg)	(g/kg)	(g/kg)	(g/kg)	(g/kg)	(Mg/ha)	(g/kg)
GKN	4.22 a	9.85 a	14.10 a	2.67 a	8.42 a	11.09 a	56.20 a	5.0 a
GN	4.30 a	9.60 a	13.72 a	2.60 a	8.50 a	11.10 a	55.76 a	4.70 a
GK	4.12 a	9.52 b	13.64 a	2.25 ab	7.52 b	9.77 b	52.23 b	3.20 b
G	4.20 a	9.50 a	13.70 a	2.22 ab	7.57 b	9.80 b	51.41 b	3.30 b
BSKN	3.72 b	8.50 b	12.37 b	1.85 b	7.32 b	9.17 b	48.05 bc	1.70 c
BSN	3.63 b	8.47 b	11.80 b	1.95 b	6.65 c	8.52 c	44.60 c	0.80c
BSK	2.80 d	7.85 c	10.60 c	2.10 ab	6.15 c	8.25 c	42.05 c	…
BS	2.55 d	7.70 c	10.22c	2.12 ab	6.57 c	8.70 c	43.62 c	…
CV	8.02	4.79	5.88	16.55	9.0	9.20	8.82	5.23

Note

Values followed by different letters in the same column indicate a significant difference at the 5% level by LSD test. GKN, Gliricidia with 60 kg/ha of K, 60 kg/ha of N; GN, Gliricidia with 60 kg/ha of N; GK, Gliricidia with 60 kg/ha of K; G, Gliricidia; BSKN, bare soil, 60 kg/ha of K, 60 kg/ha of N; BSN, bare soil 60 kg/ha of N; BSK, bare soil 60 kg/ha of K; and BS, bare soil; and CV, coefficient of variation. Ellipses indicates 5% level by LSD test.

The total accumulated N (Table 4) was increased by the addition of biomass in 2016 and 2017 (GNK = GN > BSN); however, when used alone, biomass and urea were equivalent in accumulated N (G = BSN). In turn, potassium application did not increase total organic N accumulation but increased total inorganic N accumulation when biomass was not used (BSKN > BSN). The percentage of N accumulated post‐tasseling was increased by leguminous biomass only in 2017. Except in treatments BSK and BS, where accumulated N was very small in both years, total N accumulated in 2017 was less than half the value for 2016. Differences in post‐tasseling N accumulation were even larger due to the very small amount accumulated in 2017. When comparing BSKN and GKN treatments, the increase in N provided by biomass only was 31.8 kg/ha in 2016 and 14.04 kg/ha in 2017.

**Table 4:** Accumulated nitrogen at tasseling (ANAT**)**, accumulated N post‐tasseling, accumulated N (ANPT), total accumulated N (TAN), in 2016 and 2017

Treatments	2016 (kg/ha)			2017 (kg/ha)		
	ANAT	ANPT	TAN	ANAT	ANPT	TAN
GKN	58.46 a	44.67 a	103.14 a	38.47 a	15.64 a	50.12 a
GN	64.01 a	37.99 ab	102.01 a	31.73 b	14.71 a	46.44 a
GK	29.36 b	38.03 ab	59.40 c	15.80 d	11.94 b	28.12 c
G	30.78 b	39.95 ab	70.74 b	21.51 c	8.80 c	30.32 c
BSKN	37.13 b	34.20 b	71.34 b	23.82 c	12.22 ab	36.04 b
BSN	33.66 b	30.62 bc	64.28 c	22.51 c	11.11 b	28.29 c
BSK	16.84 c	19.66 d	36.50 d	12.23 e	8.31 c	23.54 d
BS	14.74 c	21.47 cd	36.22 d	12.38 de	8.68 c	21.07 d
CV	17.81	22.89	9.83	8.62	35.66	10.68

Note

Values followed by different letters in the same column indicate a significant difference at the 5% level by LSD test. GKN, Gliricidia with 60 kg/ha of K, 60 kg/ha of N; GN, Gliricidia with 60 kg/ha of N; GK, Gliricidia with 60 kg/ha of K; G, Gliricidia; BSKN, bare soil, 60 kg/ha of K, 60 kg/ha of N; BSN, bare soil 60 kg/ha of N; BSK, bare soil 60 kg/ha of K, and BS, bare soil.

### Maize grain yield and nitrogen‐use efficiency

**Figure 3:**
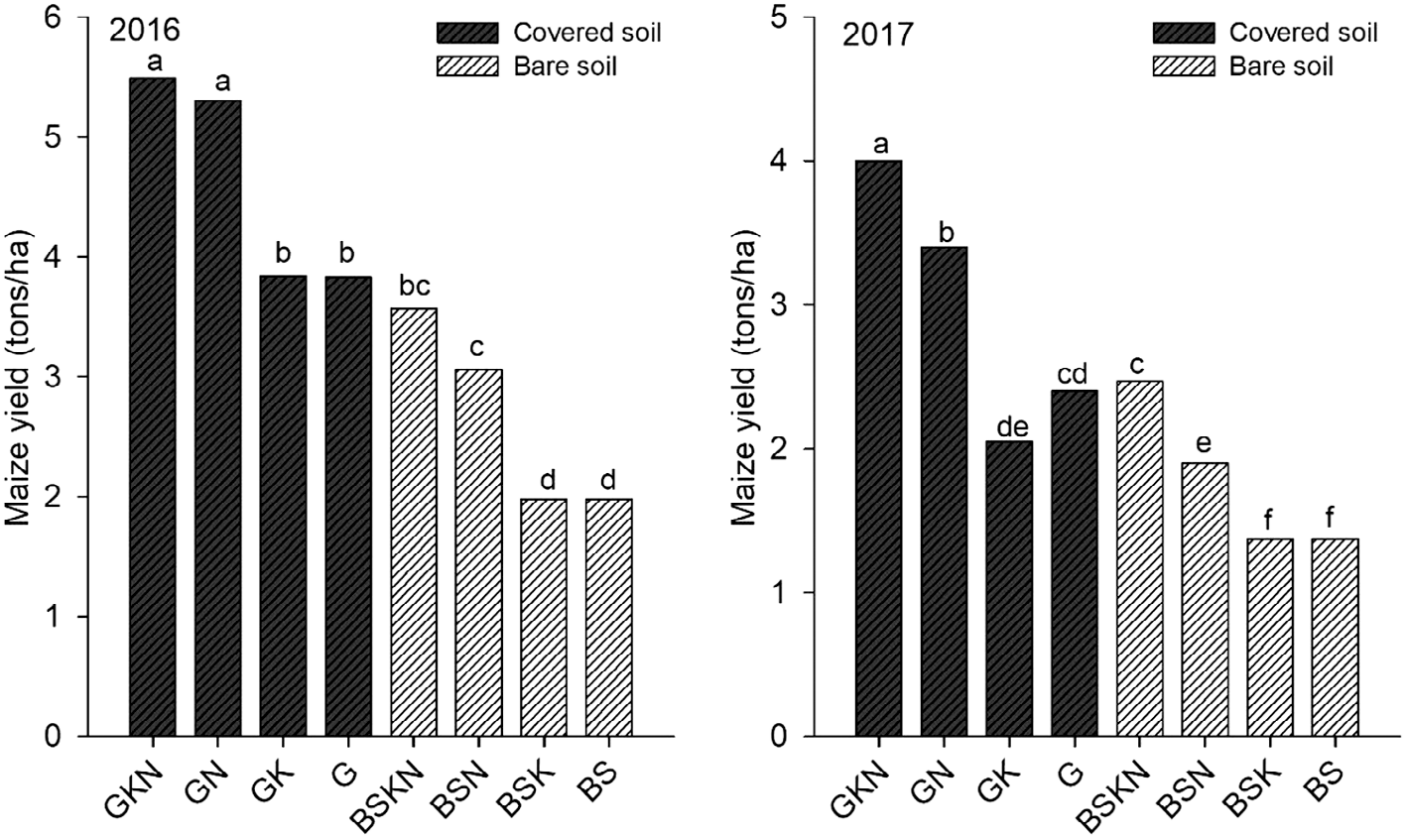
Maize grain yield in 2016 and 2017 in tons/ha.

Biomass and N application increased maize grain yield in 2016 and 2017 (Fig. 3), with and without the addition of potassium (GKN > BSKN, GN > BSN, BSKN > BSK, and BSN > BS). However, compared with treatments without biomass (BSKN and BSN), grain yield was increased to approximately 70% in the treatments with biomass (GKN, GN). In addition, grain yield was lower in treatments without biomass; differences to the control were also small (54% in 2016 and 37% in 2017). In contrast, application of potassium did not increase the maize grain yield in 2016 but did increase the yield in 2017 (GKN > GN and BSKN > BSN); therefore, compared with the control, the percentage of yield increased by BSN in 2017 was lower than the value in 2016 (59%), and the difference between GKN and BSN was 110% higher. When compared with BSKN, the increase in grain yield provided by biomass only was 1.92 kg/ha in 2016 and 1.53 kg/ha in 2017.

**Figure 4:**
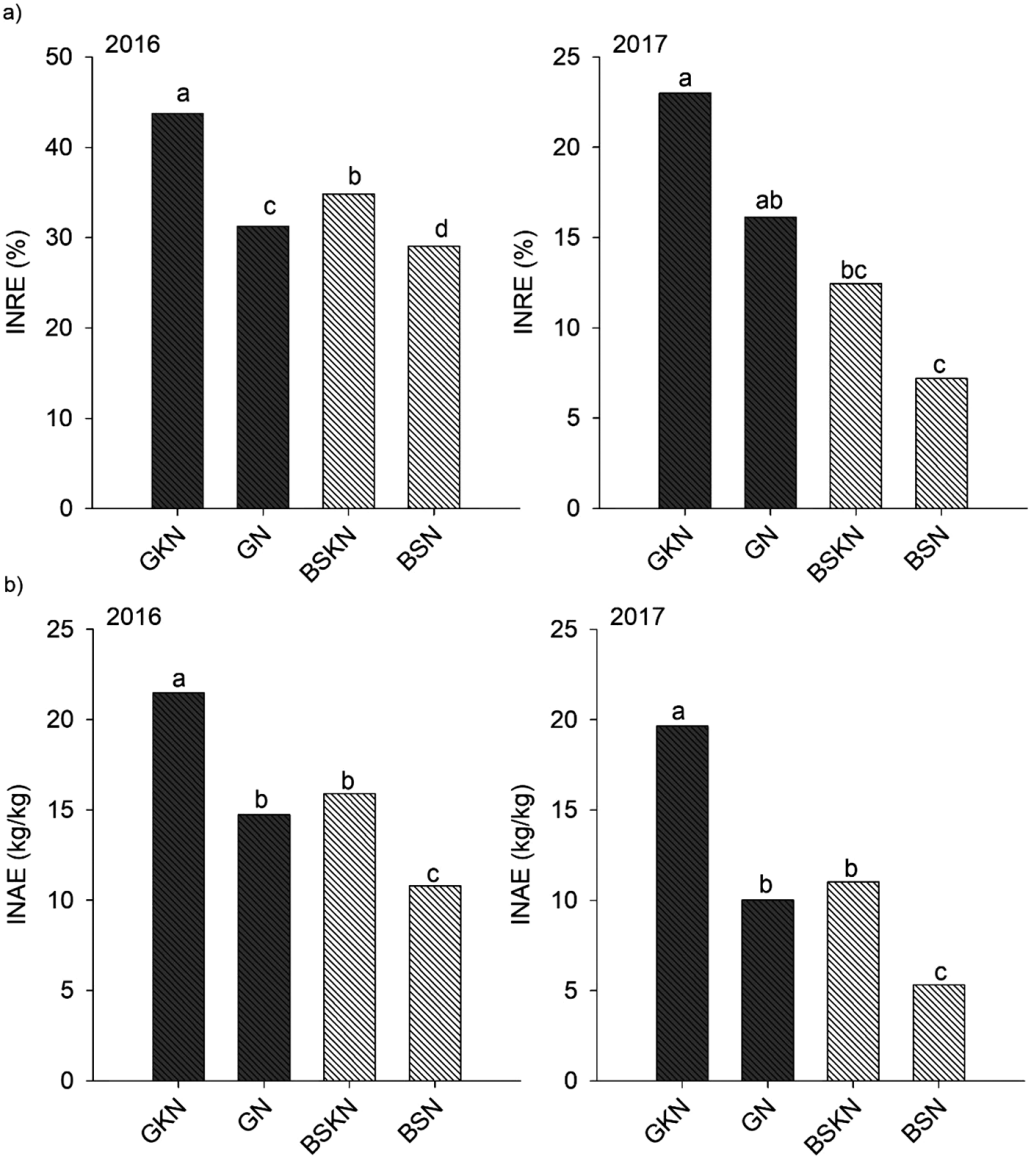
Inorganic nitrogen recovery efficiency and inorganic nitrogen agronomic efficiency in maize in 2016 and 2017.

In 2016, both biomass and potassium increased the inorganic nitrogen recovery efficiency (INRE; Fig. 4) in such a way that the difference of INRE between GKN and BSN was 52% (73.0–48.4%). In 2017, the INRE was around half of the value in 2016, and the difference between GKN and BSN was even greater (200%; 36.6–12.0%). In the same way, inorganic nitrogen agronomic efficiency (INAE) was increased by biomass and potassium in 2016 and 2017. In 2017, the INAE of bare soil treatment with N (BSN) was as low as 8.8 kg of grain per kg of N applied. Relative contribution from legumes to total N uptake was greater in GN than in GKN.

## Discussion

### Improvement in soil fertility

Our findings showed that was a positive effect of the biomass of Gliricidia on permanence of both organic carbon and base cations in the root zone. These results confirm our hypothesis that favorable tropical factors can contribute to establishment and management of sustainable integrated crop–tree system, overcoming the adversities through ecosystem services. In tropical soil, the reduction in organic carbon and loss of base cations are the main causes of land degradation and together affect soil rootability, nutrient availability, and uptake, and, consequently, the biomass and crop productivity (Basamba et al. 2006).

Although conditions in the humid tropics favor the fast decay of applied biomass, total stock of carbon was 30% higher in treatments with biomass and nitrogen than in treatments of bare soil without N. As a result, the treatments with biomass and nitrogen together increased organic matter content by 5 g/kg in just 5 yr of biomass application. The results of this experiment shown that interactions between negatively charged organic matter functional groups and polyvalent base cations can build organic compounds that are not easily reversed, preventing biological, chemical, or physical breakdown, as has been previously reported by Moore and Turunen (2004). In addition, Ellerbrock and Gerke (2018) showed that the interactions between content of adsorbed calcium and organic compounds explained approximately 83% of the organic carbon in soil. According to Nciizah and Wakindiki (2012), hardsetting soils with less than a critical concentration of SOM are likely to breakdown. Similarly, Mullins (1999) suggested a threshold of 2% SOM, below which the soil would be vulnerable to cohesion and degradation. Our finding suggests a possible mechanism for avoiding risks of land degradation by increasing ¼ of the critical level of SOM in five years, even though SOM content is highly reliant on soil management practices (Hijbeek et al. 2018). In turn, the positive effect of nitrogen on soil organic carbon can be traced to increased net primary production improving vigor of plants and gives a boost to SOM turnover. Thus includes the increase those components below ground, which, according to Russell et al. (2009), can influence the amount and decomposition rate of organic matter.

Furthermore, the higher sum of base cations achieved in our experimental treatments with biomass can be attributed to at least two processes: nutrient recycling and retention of cations by organic matter. Aguiar et al. (2010) outlined the capacity of leguminous trees to recycle nutrients and buffer the level of calcium in the root zone. However, Yamashita et al. (2008) suggested avoiding the use of a fast‐growing nitrogen fixer such as *A. mangium* because the base cations that rapidly accumulate in its biomass are matched by the extrusion of H+ from its roots. This process may lead to soil acidification and reduction of calcium by leaching with nitrate anions, which are present in high concentrations in soils where nitrogen‐fixing trees grow (Binkley and Giardina 1997). In contrast, other authors have also confirmed the positive effect of the Gliricidia biomass on increasing particulate organic matter and cation exchangeable capacity (Beedya et al. 2010) and total base cations (Moura et al. 2012).

As for cation retention, in the same way that organic matter can be maintained in soil by polyvalent cations, the formation of cation bridges between calcium and magnesium with compounds derived from decomposition of the applied biomass can explain the higher contents of these cations in treatments with biomass and nitrogen. Therefore, factors which increase organic matter also contribute to differences of sum of the base cations between the treatments GN and SN (Whittinghill and Hobbie 2012). Together, these processes can improve soil rootability and enhance nutrient uptake. Indeed, the differences in soil penetration resistance between treatments with and without biomass were remarkable. In our experiment, the small variation in soil moisture and the large difference in penetration resistance in the 10–20 cm layer suggest that factors other than soil moisture, such as calcium or organic matter, may have contributed to a reduction in penetration resistance. Both organic matter fractions and calcium are important contributors to enhanced soil rootability. The contribution of calcium results from both direct improvements (increasing flocculation and aggregation in the subsoil) and indirect improvements (enhancing root activity, which leads to greater soil aggregation; Sumner 2009). Organic matter fractions MOC and POC may reduce soil strength by increasing resistance to deformation and/or by increasing elasticity through rebound effects. Thus, soil compatibility is sensitive to even small changes in the amount of organic matter mainly to mineral fraction because many of the long‐chain molecules this fractions are very effective at binding mineral particles Soane (1990). These observations have important environmental implications when considering how to improve cohesive soil management for root growth, nutrient use efficiency, and even agroecosystem production.

### Use of nitrogen and maize grain yield

Differences in N uptake can be attributed to two processes: enhancement in soil rootability which could lead to enhanced root growth and increased N availability, both of which were positively influenced by biomass application. N uptake is highly reliant on root size and architecture, which must be important variables in managing soil for N acquisition across the soil profile (Dechorgnat et al. 2018). In addition, the elongation rate of roots is highly correlated with the penetration resistance of uniform soil, such that roots growing in hard soil are morphologically different from those growing in friable soil (Passioura 1991). On the other hand, the equivalence between G and BSKN treatments showed the importance of using biomass as green manure in humid tropic conditions, for improving soil water as much as for providing N. Furthermore, when compared with BSN and G treatments, the increase of almost 50% achieved by GN treatments suggests that the combined use of green manure and synthetic fertilizers may be a better strategy for enhancing N uptake, as was suggested by Espinal et al. (2016). A higher precipitation rate in 2017 decreased N uptake in that year. As the flux of soil mineral N is largely influenced by precipitation events, the N losses through leaching may increase with increased precipitation. Increased N leaching might also reduce N available for crop growth (Jabloun et al. 2015). Therefore, it was only in 2017 that the percentage of N uptake post‐tasseling was increased by the addition of leguminous biomass. Without biomass addition, potassium fertilization increased N uptake. Authors such as Santa‐Maria et al. (2000) have reported a protective role of adequate K+ concentration against the detrimental effects of high NH_+4_ concentrations. Other authors such as Khalifa et al. (2012) have also suggested that K+ in plant roots could counteract a possible toxic effect of ammonium nutrition, enhancing ammonium utilization. It is concluded that K+ may alleviate NH_+4_ toxicity, partly by inhibiting NH_+4_ uptake, partly by stimulating N assimilation in the roots, and decreasing its volatilization.

According to Wolz and DeLucia (2019), alley cropping has shown great potential as a scalable agricultural alternative that can enhance production while simultaneously improving sustainability in the Midwest United States. Our results from alley cropping in the humid tropics of Amazonian periphery also showed that the biomass of Gliricidia had a positive effect on soil improvement and nitrogen uptake, thereby contributing to agricultural intensification by increasing maize grain yield by 3.5 tons/ha. In bare soil, the additional yield of 1.5 tons/ha would not be enough to convince farmers to change slash and burn to conventional bare soil systems. This assessment was best evidenced by the nitrogen agronomic efficiency in 2016 and 2017 when comparing the BSN treatment (18 and 8 kg of grain per kg of applied N) with the GN treatment (24 and 16 kg of grain per kg of applied N). Higher rainfall precipitation in 2017 decreased grain yield and increased the importance of potassium to NRE and NAE when biomass was not used. The effect of biomass and potassium on NRE and NAE was made explicit by comparison with other treatments, confirming the importance of N management for agroecosystem success in the humid tropics.

## Conclusions

Leguminous trees such as Gliricidia might contribute to achieving sustainable agricultural intensification in soil with low natural fertility, as is characteristic of the humid tropics, by providing ecosystem services such as biomass production, carbon sequestration, base cation recycling, and increased N acquisition. The contribution of Gliricidia biomass to changes in the root zone was significant in terms of increasing soil fertility and nitrogen uptake. In just five years of biomass application, the soil organic matter was increased by 5.0 g/kg, and the sum of base cations was increased by 645 kg/ha in the 0–30 cm layer. Moreover, grain yield was increased by approximately 70% due to increased nitrogen uptake and use efficiency.

Moreover, grain yield was increased by approximately 70% in the treatments with *Gliricidia* (4.50 tons in average) when compared to treatments without biomass where yield was very low (2.64 tons in average). These findings might be part of an important strategy to replace the slash‐and‐burn‐system and preserve the rainforest against the traditional shifting cultivation system. In contrast, the conventional system soil showed that in bare soil the additional yield of 1.5 tons/ha with the use of nitrogen would not be enough to convince farmers to change slash and burn to conventional bare soil systems. This could be truly unfeasible, mainly when in conditions of high rainfall precipitation. In these circumstances, the use of potassium may increase nitrogen‐use efficiency only when biomass is not used.
